# Antiproliferative and Antimetastatic Properties of 16-Azidomethyl Substituted 3-*O*-Benzyl Estrone Analogs †

**DOI:** 10.3390/ijms241813749

**Published:** 2023-09-06

**Authors:** Seyyed Ashkan Senobar Tahaei, Ágnes Kulmány, Renáta Minorics, Anita Kiss, Zoltán Szabó, Péter Germán, Gábor J. Szebeni, Nikolett Gémes, Erzsébet Mernyák, István Zupkó

**Affiliations:** 1 Institute of Pharmacodynamics and Biopharmacy, University of Szeged, H-6720 Szeged, Hungary; 2 Department of Inorganic, Organic and Analytical Chemistry, University of Szeged, H-6720 Szeged, Hungary; 3 Department of Medicinal Chemistry, University of Szeged, H-6720 Szeged, Hungary; 4 Laboratory of Functional Genomics, Biological Research Centre, H-6726 Szeged, Hungary; 5 Interdisciplinary Centre of Natural Products, University of Szeged, H-6720 Szeged, Hungary

**Keywords:** estrone analogs, antiproliferative effect, metastasis, apoptosis, tubulin polymerization, breast cancer

## Abstract

Four diastereomers of 16-azidomethyl substituted 3-*O*-benzyl estradiol (**1**–**4**) and their two estrone analogs (**16AABE** and **16BABE**) were tested for their antiproliferative properties against human gynecological cancer cell lines. The estrones were selected for additional experiments based on their outstanding cell growth-inhibiting activities. Both compounds increased hypodiploid populations of breast cancer cells, and **16AABE** elicited cell cycle disturbance as evidenced by flow cytometry. The two analogs substantially increased the rate of tubulin polymerization in vitro. **16AABE** and **16BABE** inhibited breast cancer cells’ migration and invasive ability, as evidenced by wound healing and Boyden chamber assays. Since both estrone analogs exerted remarkable estrogenic activities, as documented by a luciferase reporter gene assay, they can be considered as promising drug candidates for hormone-independent malignancies.

## 1. Introduction

Cancer is prominent cause of death worldwide, with mortality rates comparable to those of stroke and coronary heart disease. According to the latest update from the International Agency for Research on Cancer (IARC) database, 19.3 million new cancer cases and almost 10 million cancer-related deaths occurred globally in 2020. Moreover, the global cancer burden is expected to reach 28.4 million cases by 2040, corresponding to a 47% rise in all cancer cases. Lung cancer is the leading cause of cancer death, responsible for 18% of tumor-related mortality, followed by colorectal (9.4%), liver (8.3%), stomach (7.7%), and female breast (6.9%) cancers. Concerning the incidence of different female tumors, breast carcinomas are the most common, accounting for 24.5% of all new cases. Altogether, 38.9% of new cancer cases in females involve gynecological malignancies [1]. The 2.26 million breast cancer cases diagnosed in 2020 show unequal geographical distribution, with the highest age-standardized incidence rate in Europe (69.7/100,000) and the lowest in South-East Asia (28.3/100,000). A statistically significant inverse correlation was observed between the mortality-to-incidence ratio (MIR) and the human development index (HDI), indicating poorer prognosis for patients living in less developed regions of the world [2]. All these epidemiological findings suggest that the prevention and treatment of female breast cancers are not yet resolved, despite the impressive therapeutic progress evidenced in past decades.

Substantial improvement of the global cancer burden is impossible without innovative therapeutic options, including original drugs. Studying compounds with steroidal skeleton as potential anticancer agents has a long history. Over the past few decades, several new steroids, such as cyproterone, finasteride, exemestane and fulvestrant, have been integrated into clinical practice. A feasible strategy for developing novel drug candidates involves the chemical modification of endogenous molecules to produce semi-synthetic analogs with diverse biological activities [3].

The initial application of steroid-based compounds in the field of anticancer therapy emerged from the utilization of diverse botanical extracts. Evidence suggests that steroid-like triterpenes, including betulinic acid, oleanolic acid, and related derivatives, exhibit potent proapoptotic and antimigratory effects against numerous human cancer cell lines [4,5,6,7,8,9,10]. Many estrane-based compounds modified in rings A or D have been investigated recently, demonstrating that triazolyl estranes exert promising anticancer actions [11,12]. Studies have also demonstrated that numerous core-modified estradiol analogs exhibit considerable antiproliferative activity against human cancer cell lines derived from gynecological malignancies [13]. The position, specific nature, size and polarity of the substituents newly introduced into the molecule have been shown to substantially impact the anticancer properties of the designed derivatives. The antiproliferative mechanism of certain core-modified estrones is based on their direct effects on the tubule-microtubule system, resulting in disturbed tubulin polymerization rates [11,14,15].

We have recently reported on certain 16,17-functionalized 3-methoxy or 3-benzyloxy estrone derivatives behaving as potent antiproliferative compounds [16,17]. The substitution pattern of ring D, and the nature of the protecting group at C-3-*O* was demonstrated to influence the cell growth-inhibitory potential of these compounds markedly. Overall, 3-benzyl ethers were found to be more potent [16]. The substituents’ nature and orientation affected the antitumoral behavior of these previously tested agents [17].

**Figure 1:**
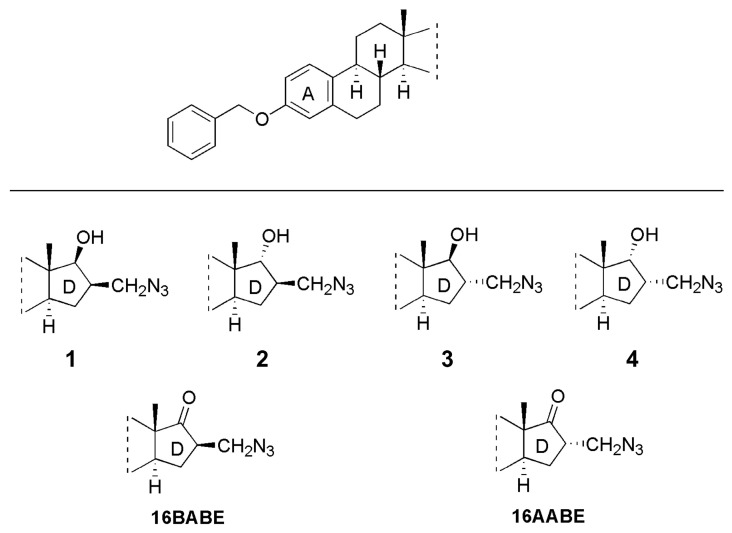
Structures of the tested starting compounds (**1**–**4**) and the newly synthesized agents 16β-azidomethyl-3-*O*-benzyl estrone (**16BABE**) and 16α-azidomethyl-3-*O*-benzyl estrone (**16AABE**).

Based on these promising findings regarding the antiproliferative activities of 16,17-functionalized estrone 3-benzyl ethers, in the present study we aimed to assess the antiproliferative, antimetastatic and anticancer properties of these novel substituted steroidal compounds, including four 16-azidomethyl-17-hydroxy derivatives (**1**–**4**) and their 17-keto counterparts (**16AABE** and **16BABE**, Figure 1).

## 2. Results

### 2.1. Chemistry

**Scheme 1:**
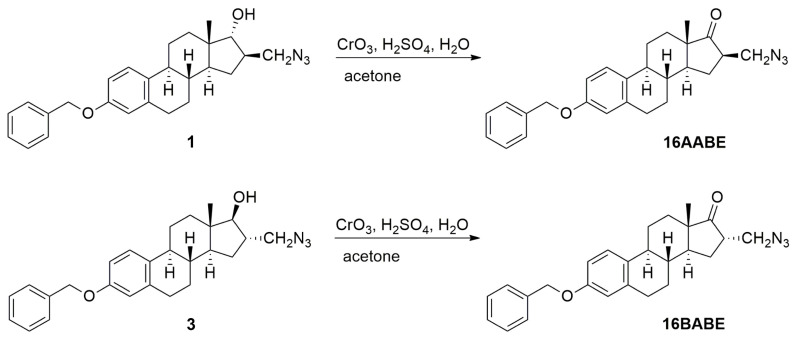
Syntheses of **16BABE** and **16AABE**.

Compounds **16AABE** and **16BABE** were synthesized from their 17-hydroxy precursors (1 and 3, Scheme 1). The starting compounds were subjected to oxidation using the Jones reagent. The reactions furnished the products **16AABE** and **16BABE** in high yields. The structures of 17-keto compounds were deduced from ^1^H and ^13^C NMR assessments.

### 2.2. Antiproliferative Assay

**Figure 2:**
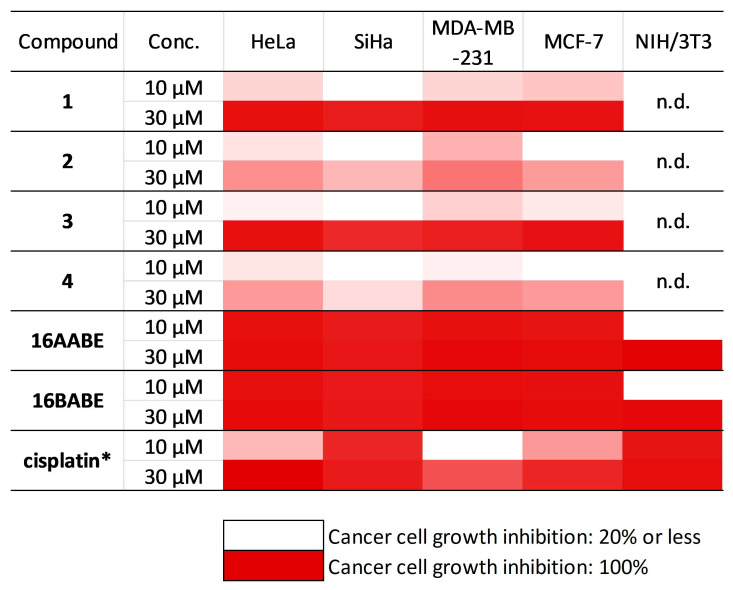
Antiproliferative properties of the investigated molecules. Inhibition values <20% are considered negligible and are not given numerically. n.d.: not determined. *: data are from refererence [17]. Numeric results with calculated IC_50_ values are presented in Supplementary Table S1.

The antiproliferative capacities of the prepared compounds were determined by employing the MTT assay against a panel of human adherent cancer cell lines isolated from breast (MCF-7 and MDA-MB-231) or cervical (HeLa and SiHa) tumors. All compounds were tested at two concentrations (10 and 30 μM). When >50% of antiproliferative capacity was obtained at 10 μM, the assays were repeated with a broader concentration range (0.1–30 μM), and IC_50_ values were calculated (Figure 2, Supplementary Table S1, Supplementary Figure S1). Starting molecules **1**–**4** exerted negligible action at 10 μM, but substantial cell growth inhibition was observed at the higher concentration (30 μM). On the other hand, the 17-keto analogs (**16AABE** and **16BABE**) elicited over 90% inhibition even at the lower concentration, and their calculated IC_50_ values were lower than that of the reference agent cisplatin. MTT assays were performed against the non-cancerous fibroblast cell line NIH/3T3 to obtain preliminary data on cancer selectivity of **16AABE** and **16BABE**. The fibroblast cells proved to be less sensitive, with calculated IC_50_ values >10 μM. The ratios of IC_50_ values obtained against cancer cells and fibroblasts were in the range of 0.2 and 0.5, indicating substantial cancer selectivity of these two compounds (Table 1).

### 2.3. Propidium Iodide-Based Cell Cycle Analysis

**Figure 3:**
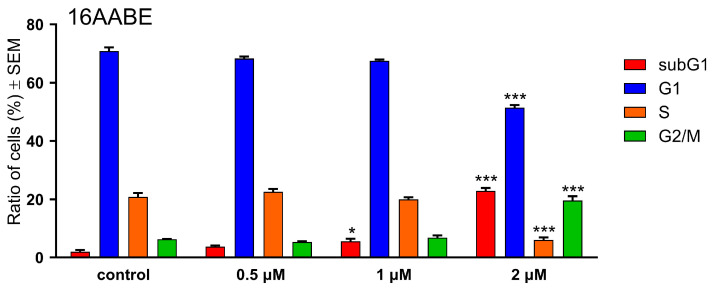

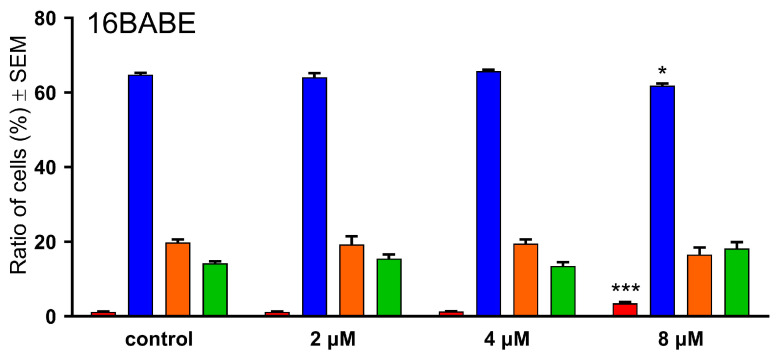
Effects of **16AABE** (upper panel) and **16BABE** (lower panel) on cell cycle distribution of MDA-MB-231 cells treated with the indicated concentrations for 24 h. * and *** indicate significant differences at *p* < 0.05 and *p* < 0.001, respectively. Data are from three independent experiments performed in triplicate.

**Figure 4:**
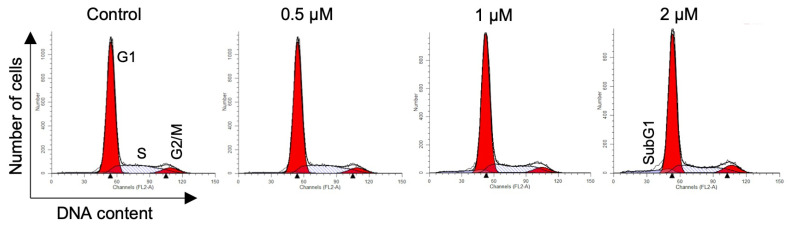
Representative histograms for MDA-MB-231 cells treated with **16AABE**. Histograms were generated using the ModFit LT 3.3.11 software.

**16AABE** and **16BABE** were subjected to propidium iodide-based cell cycle analysis by flow cytometry to elucidate their mechanism of action. MDA-MB-231 cells were treated with various concentrations of the test agents for 24 h, and DNA content of the cells was determined. **16AABE** induced a moderate but significant increase in the hypodiploid (subG1) cell population at 1 μM (Figure 3 and Figure 4). At 2 μM, which approximately equals the IC_50_ value for this agent, a more profound cell cycle disturbance was observed with a pronounced increase in the subG1 and G2/M populations at the expense of G1 and S phases. Conversely, **16BABE** induced a minor but significant accumulation of subG1 cells at 8 μM, a concentration roughly equaling its IC_50_, indicating the proapoptotic activity of this compound (Figure 3).

### 2.4. Tubulin Polymerization Assay

**Figure 5:**
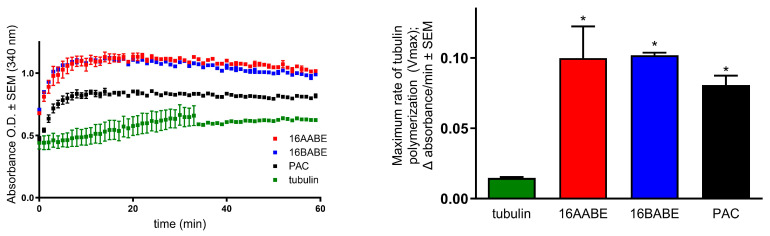
Direct effects of **16AABE** and **16BABE** (500 μM for both) on tubulin polymerization. Left panel: recorded kinetic curves; paclitaxel (10 μM PAC) was included as a reference agent. Right panel: calculated maximum values for the rate of tubulin polymerization. * indicates significance at *p* < 0.05 compared with untreated control. Data are from two independent experiments performed in duplicate.

The impact of **16AABE** and **16BABE** on microtubule polymerization was assessed using a cell-free system with a photometric kinetic determination. Concentrations of the test compounds were selected based on their IC_50_ values, as recommended by the kit’s manufacturer. Both compounds exhibited a stimulating effect on tubulin polymerization compared with control. Notably, the calculated maximum rates of tubulin polymerization (Vmax) were significantly higher than those observed for the control (Figure 5). Additionally, the Vmax values for the test compounds were higher than that for the reference agent paclitaxel (PAC, 10 μM), indicating their profound activity on tubulin polymerization.

### 2.5. Wound Healing Assay

**Figure 6:**
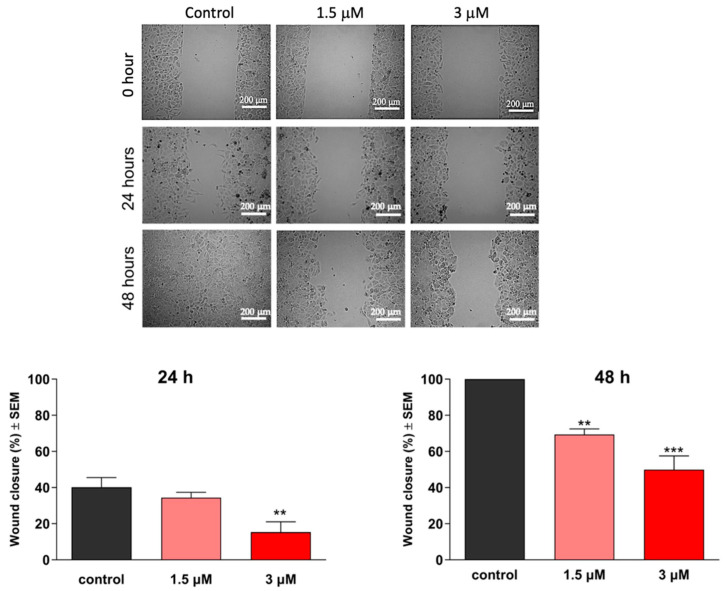
Effects of **16AABE** on the migration of MCF-7 cells. Upper panels: representative images taken at 24 or 48 h post-treatment with **16AABE**. Lower panels: calculated wound closure values determined at 24 or 48 h post-treatment. ** and *** indicate significance at *p* < 0.01 and *p* < 0.001, respectively. Data are based on 4 independent experiments, all performed in triplicate.

**Figure 7:**
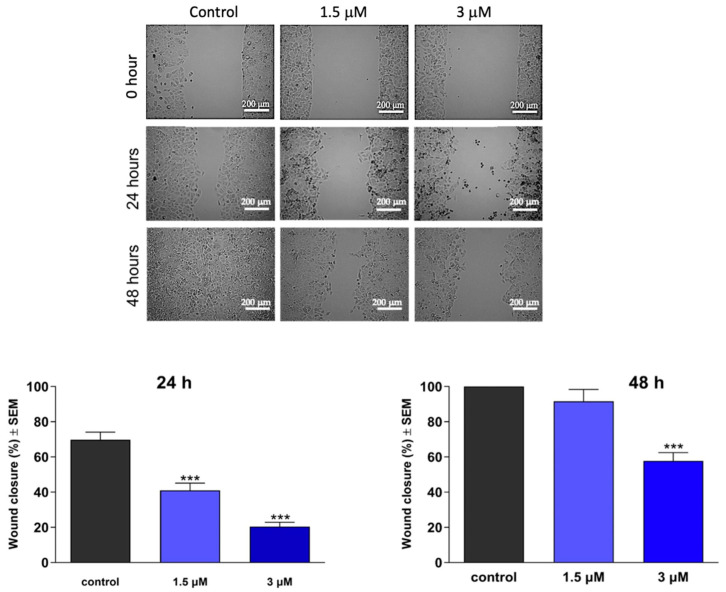
Effects of **16BABE** on the migration of MCF-7 cells. Upper panels: representative images taken at 24 or 48 h post-treatment with **16BABE**. Lower panels: calculated wound closure values determined at 24 or 48 h post-treatment. *** indicates significance at *p* < 0.001. Data are based on 4 independent experiments, all performed in triplicate.

To investigate the antimigratory activity of the test compounds, we conducted a wound-healing assay using the MCF-7 breast cancer cell line. Using an in vitro model of wound closure, a wound was created by removing silicone inserts from a cell-covered chamber, followed by incubating the cells in a minimal serum-containing (2%) medium for 0, 24, and 48 h. Microscope image analysis was performed to measure the reduction in cell-free areas, serving as an indicator of wound closure. Our findings demonstrated a significant decrease in the migratory capacity of cancer cells (Figure 6 and Figure 7). Notably, both compounds exhibited remarkable antimigratory effects at subantiproliferative concentrations (1.5 μM), with **16BABE** demonstrating a more pronounced action after 24 h of incubation.

### 2.6. Boyden Chamber Assay

**Figure 8:**
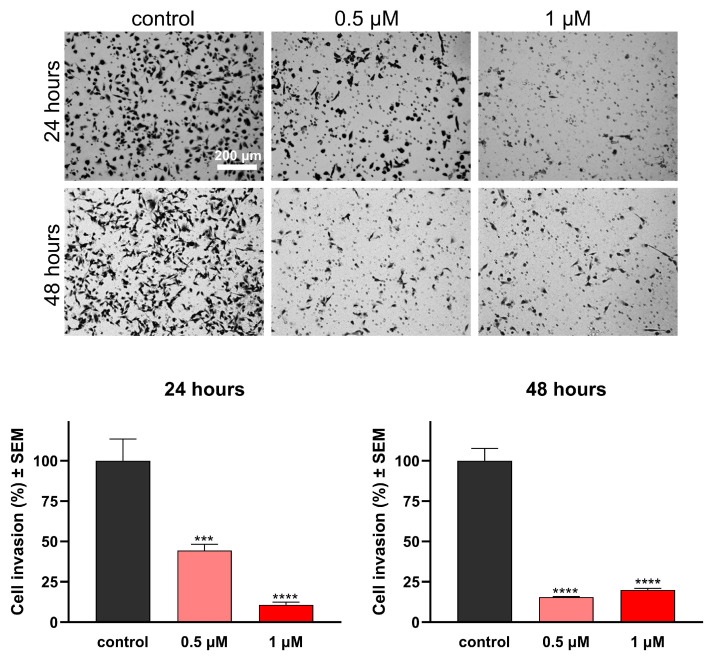
Effects of **16AABE** on the invasion capacity of MBA-MD-231 cells. Upper panels: representative images taken at 24 or 48 h post-treatment with **16AABE**. Lower panels: **16AABE** significantly reduced invasion of MDA-MB-231 cells at 24 h and 48 h post-treatment. Data are based on at least 4 independent experiments performed in duplicate. *** and **** indicate significance at *p* < 0.001 and *p* < 0.0001, respectively.

**Figure 9:**
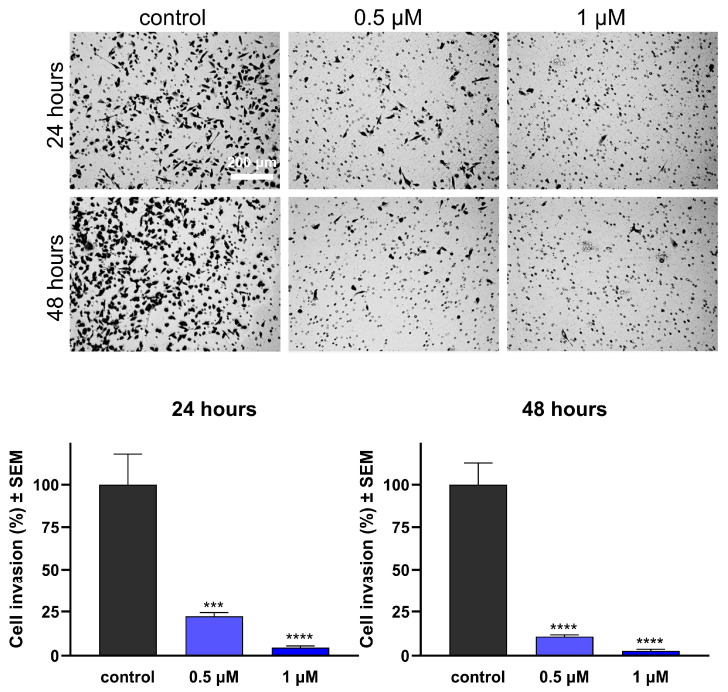
Effects of **16BABE** on the invasion capacity of MBA-MD-231 cells. Upper panels: representative images taken at 24 or 48 h post-treatment with **16BABE**. Lower panels: **16BABE** significantly reduced the invasion of MDA-MB-231 cells at 24 h and 48 h post-treatment. Data are based on at least 4 independent experiments performed in duplicate. *** and **** indicate significance at *p* < 0.001 and *p* < 0.0001, respectively.

As the invasive capacity of cancer cells plays a pivotal role in metastatic behavior, it is crucial to assess the antimetastatic potential of any promising anticancer agents, in addition to characterizing their impact on cell migration. Boyden chambers with Matrigel Matrix-coated membranes (pore diameter: 8.0 μm) were employed to evaluate invasiveness, as they permit the passage of invasive cells while impeding the migration of non-invading cells. Remarkably, the test compounds hindered the invasion of MDA-MB-231 cells efficiently, even at low concentrations of 0.5 or 1 μM at 24 h post-treatment (Figure 8 and Figure 9). Moreover, both compounds exhibited a significant decrease in invading cells after 48 h of treatment, supporting their remarkable anti-invasive potential.

### 2.7. Estrogenic Activities of the Test Compounds

**Figure 10:**
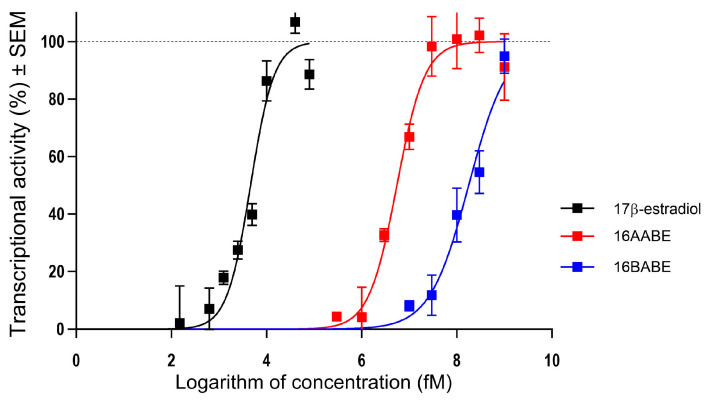
Estrogenic effects of **16AABE** and **16BABE** expressed as the intensity of the estrogen-responsive luciferase in transfected T47D breast cancer cell line. Data are based on 3 independent experiments performed in triplicate.

Since **16AABE** and **16BABE** are structurally closely related to the natural estrogen 17β-estradiol, their hormonal activities are considered crucial elements of their pharmacological profile. A T47D breast cancer cell line transfected with an estrogen-responsive luciferase reporter gene was utilized to clarify the estrogenic activity of the test compounds (Figure 10). Both agents were found to exert estrogenic activity at concentrations several orders of magnitude higher than the reference agent 17β-estradiol. The calculated concentrations eliciting 50% of maximum estrogenic stimulation were approximately 5.5 nM and 178 nM, respectively. These results indicate that the tested estrone analogs possess considerable hormonal activity at their antiproliferative or antimetastatic concentrations.

## 3. Discussion

Breast cancer is the most frequent malignancy in females globally. Based on crucial molecular markers, including estrogen and progestin receptors and human epidermal growth factor receptor 2 (HER2), the disease entity is classified into major subtypes: hormone receptor (HR) positive, HER2-positive, and triple-negative breast cancers (TNBC). TNBC accounts for approximately 15–20% of all cases, and its prevalence seems to be higher in younger patients, below 40 years of age [18,19]. TNBC exhibits aggressive behavior compared with other subtypes, and has a poorer prognosis. Due to the lack of targeted pharmacological interventions, current treatment of TNBC is limited to traditional cytotoxic agents [20].

Although estrogens, including the natural hormone 17β-estradiol, are generally considered to promote cell growth, several estrane-based molecules have been identified as potent anticancer drug candidates [13].

The 16-substituted triazolyl estranes represent a class of compounds with a unique structural framework combining a triazole ring with an estrane scaffold. The design and synthesis of these compounds involve click chemistry and structure-activity relationship studies to optimize their pharmacological profiles. Continued research and optimization of 16-substituted triazolyl estranes hold promise for developing novel therapeutics across multiple disease areas [11].

Our current study has focused on investigating the antiproliferative properties of four 16-azidomethyl estradiol analogs (**1**–**4**) previously utilized as intermediaries in synthesizing 16-triazolyl estranes [17]. Our screens for antiproliferative activity were extended to cover two estrone congeners (**16AABE** and **16BABE**), and compounds with a 17-keto function were found to be more active than the reference agent cisplatin. Moreover, the ratios of IC_50_ values obtained against cancer cells and NIH/3T3 fibroblasts were below 1 (within the range of 0.166 and 0.429), indicating reliable cancer selectivity. At the same time, 17-hydroxy analogs exhibited modest actions only. According to the calculated IC_50_ values, the stereochemical difference, i.e., the configuration of the 16-azidomethyl group, is not a crucial factor in the activity of these compounds. Based on these findings, the two estrone analogs were subjected to additional investigations to characterize their anticancer activities in detail.

Cell cycle analysis generally provides valuable insights into the mechanisms responsible for disturbing cell proliferation. Both selected compounds, **16AABE** and **16BABE** induced cell cycle disturbance in MDA-MB-231 TNBC cells. Treatment with **16AABE** resulted in a concentration-dependent increase in the hypodiploid (subG1) population after 24 h of incubation. This action was detected at concentrations below the IC_50_ (1 and 2 μM), indicating the proapoptotic potency of this compound [21]. Additionally, profound accumulation of cells in the G2/M phase at the expense of the G1 and S populations was observed. On the other hand, **16BABE** elicited a detectable change in cell cycle distribution at its IC_50_ only (8 μM), and this action was limited to a modest increase in the subG1 and a decrease in the G1 cell population.

Based on these cell cycle disturbances, investigations into the effects on tubulin polymerization seemed rational. Microtubules, these highly dynamic filamentous proteins within the cytoskeleton, are considered significant targets for anticancer interventions [22]. Both compounds were found to induce a considerable increase in tubulin polymerization rate at a concentration of 500 μM, indicating their ability to enhance microtubule assembly and stability. These effects were comparable or even superior to that of the positive control paclitaxel, highlighting that direct action on tubulin seems to be a crucial component of our test compounds’ pharmacological profile.

According to epidemiological data, approximately 90% of cancer-related deaths can be attributed to metastases [23]. This complex sequence of events encompasses several stages, including the local migration and invasion of tumor cells into neighboring tissues, penetration into the vascular system, survival, and exit from the circulatory system, followed by proliferation in distant organs, resulting in the establishment of new colonies [24]. Epidemiological evidence highlights the significant prevalence of invasive cervical cancer, ranking the fourth most frequent female malignancy after breast, colon, and lung cancers globally. Metastases of cervical carcinomas occur through either the hematogenous or lymphatic pathways. Patients with hematogenous metastases generally exhibit lower survival rates than those with lymphatic metastases [25,26,27,28]. These epidemiological characteristics illustrate the importance of developing effective antimetastatic compounds as potential drug candidates to hinder these tendencies.

Both **16AABE** and **16BABE** exhibited significant inhibitory effects on the migration of MDA-MB-231 cells. Both compounds demonstrated time- and concentration-dependent inhibition of cell migration as evidenced by the wound healing assay. Moreover, this antimigratory action was detected at a concentration of 1.5 μM, much lower than the IC_50_ values for cell growth inhibition in any cell lines tested. Based on these findings, the antimigratory properties of the test compounds may be explained by a separate pharmacological mechanism, rather than a consequence of cell growth inhibition.

A Boyden chamber assay was employed to evaluate the anti-invasive properties of our estrone analogs. After 24 h of treatment, both compounds demonstrated highly significant inhibition of breast cancer cell invasion at concentrations of 0.5 μM and 1 μM. Their actions became even more pronounced after 48 h of incubation. The exact characterization of the mechanism of their antimetastatic activities is beyond the scope of this study. However, in a previous study we investigated a set of 3-*O*-sulfamoyl-13α-estrone derivatives, and their pharmacological profile showed features similar to these currently tested compounds [29]. In that series, molecular docking studies were performed for three 13α-estrones to elucidate their binding properties to β-tubulin, and their binding affinity was found to correlate with their positive action on tubulin polymerization. Based on these findings, β-tubulin can be suggested as the probable site of action for **16AABE** and **16BABE**.

Since the role of microtubules is not limited to constructing the mitotic spindle, a tubulin disruptor may exert additional activities besides the expected antimitotic action. As tubulin dynamics are deeply involved in the mobility of cancer cells, pharmacological interventions affecting tubulin polymerization may influence metastatic potency, independently of the direct cytotoxicity of a given agent [30].

Finally, the estrane skeleton justified the characterization of the estrogenic activity of the tested analogs. Our findings indicate that **16AABE** and **16BABE** exhibit substantial hormonal activity at concentrations required for the antiproliferative and antimetastatic actions. Since a drug with estrogenic effect may promote the proliferation of estrogen sensitive cancer cells, this characteristic seems to be disadvantageous in most gynecological cancers. However, in a subclass of hormone-independent malignancies including triple-negative breast cancer, the hormonal agonist action may not limit the usability of such an agent. Therefore, our currently presented estrone analogs can be considered as innovative drug candidates for such hormone-neutral cancerous disorders.

## 4. Materials and Methods

### 4.1. Chemistry

Melting points (Mp) were determined with a Kofler hot-stage apparatus and were uncorrected. Elemental analyses were performed with a PerkinElmer CHN analyzer model 2400 (PerkinElmer, Waltham, MA, USA). Thin-layer chromatography involved silica gel 60 F254; layer thickness 0.2 mm (Merck, Budapest, Hungary); eluent (ss): 20% ethyl acetate/80% hexane; detection with I_2_ or UV (365 nm) after spraying with 5% phosphomolybdic acid in 50% aqueous phosphoric acid and heating at 100–120 °C for 10 min. Flash chromatography involved: silica gel 60, 40–63 μm (Merck). ^1^H NMR spectra were recorded in CDCl_3_ solution with a Bruker DRX-500 instrument (Bruker, Billerica, MA, USA) at 500 MHz, with Me_4_Si as the internal standard. ^13^C NMR spectra were recorded with the same instrument at 125 MHz under the same conditions (Supplementary Figures S2 and S3). Mass spectrometry: full scan mass spectra of the compounds were acquired in the range of 50 to 1000 *m*/*z* with a Finnigan TSQ-7000 triple quadrupole mass spectrometer (Finnigan-MAT, San Jose, CA, USA) equipped with a Finnigan electrospray ionization source. Analyses were performed in positive ion mode using flow injection mass spectrometry with a mobile phase of 50% aqueous acetonitrile containing 0.1% (*v*/*v*) formic acid. The flow rate was 0.3 mL/min. Five µL aliquot of the samples were loaded into the flow. The ESI capillary was adjusted to 4.5 kV and N_2_ was used as a nebulizer gas.

The general procedure for the synthesis of 16β-azidomethyl-3-benzyloxyestra-1,3,5(10)-trien-17-on (**16BABE**) and 16α-azidomethyl-3-benzyloxyestra-1,3,5(10)-trien-17-on (**16AABE**) was as follows.

Compound **1** or **3** (417 mg, 1.00 mmol) was dissolved in acetone (5 mL), then cooled in an ice-water bath, and Jones reagent (0.4 mL, 8 N) was added in five portions. The reaction mixture was allowed to stand at room temperature for 1 h, then it was diluted with water and extracted with ethyl acetate. The combined organic phases were washed with water until neutral and dried over sodium sulfate, and the crude product was subjected to column chromatography with dichloromethane/hexane = 8/2 as eluent.

Compound **16BABE** was obtained as a white solid (382 mg, 92%). Mp 86–88 °C, Rf = 0.63. Anal. calcd. for C_26_H_29_N_3_O_2_: C, 75,15; H, 7.03. Found: C, 75, 27; H, 7.07. ^1^H NMR (500 MHz, CDCl_3_) δ ppm: 0.90 (m, 1H); 0.92 (s, 3H, 13-CH_3_); 1.27–1.63 (overlapping multiplets with hexanes solvent peaks, 16H); 1.98–2.08 (overlapping multiplets, 3H); 2.22–2.32 (overlapping multiplets, 2H); 2.40 (m, 1H); 2.90 (m, 2H, 6-H_2_); 3.62 (m, 2H, 16a-H_2_); 5.04 (s, 2H, OCH_2_); 6.74 (d, 1H, J = 2.5 Hz, 4-H); 6.79 (dd, 1H, J = 8.5 Hz, J = 2.6 Hz, 2-H); 7.20 (d, 1H, J = 8.6 Hz, 1-H); 7.32 (t, 1H, J = 7.7 Hz, 4′-H); 7.39 (t, 2H, J = 7.7 Hz, 3′- and 5′-H); 7.43 (d, 2H, J = 7.7 Hz, 2′- and 6′-H). ^13^C NMR (CDCl_3_) δ ppm: 13.4 (C-18); 25.8 (CH_2_); 26.3 (CH_2_); 26.7 (CH_2_); 29.6 (CH_2_); 31.9 (CH_2_); 37.7 (CH); 44.1 (CH); 48.3 (C-13); 48.9 (CH); 49.4 (CH); 51.6 (C-16a); 69.9 (OCH_2_); 112.4 (CH); 114.9 (CH); 126.2 (C-1); 127.4 (2C, 2× CH); 127.8 (CH); 128.5 (2C, 2× CH); 132.1 (C-10); 137.2 (C); 137.7 (C); 156.9 (C-3); 219.0 (C=O). MS *m*/*z* (%) 416 (100, [M+H]^+^).

Compound **16AABE** was obtained as a white solid (374 mg, 90%). Mp 80–82 °C, Rf = 0.63. Anal. calcd. for C_26_H_29_N_3_O_2_: C, 75, 15; H, 7.03. Found: C, 75, 22; H, 7.09. ^1^H NMR (500 MHz, CDCl_3_) δ ppm: 0.88 (m, 1H); 0.97 (s, 3H, 13-CH_3_); 1.26–1.56 (overlapping multiplets with hexanes solvent peaks, 18H); 1.91–2.00 (overlapping multiplets, 4H); 2.27 (m, 1H); 2.39 (m, 1H); 2.75 (m, 1H); 2.90 (m, 2H, 6-H_2_); 3.51–3.62 (overlapping multiplets, 2H, 16a-H_2_); 5.05 (s, 2H, OCH_2_); 6.74 (d, 1H, J = 2.5 Hz, 4-H); 6.79 (dd, 1H, J = 8.5 Hz, J = 2.6 Hz, 2-H); 7.19 (d, 1H, J = 8.6 Hz, 1-H); 7.32 (t, 1H, J = 7.7 Hz, 4′-H); 7.38 (t, 2H, J = 7.7 Hz, 3′- and 5′-H); 7.43 (d, 2H, J = 7.7 Hz, 2′- and 6′-H). ^13^C NMR (CDCl_3_) δ ppm: 14.4 (C-18); 25.8 (CH_2_); 26.0 (CH_2_); 26.4 (CH_2_); 29.6 (CH_2_); 31.4 (CH_2_); 38.3 (CH); 43.9 (CH); 44.4 (C-13); 48.2 (CH); 48.6 (C-13); 51.8 (C-16a); 70.0 (OCH_2_); 112.4 (CH); 114.9 (CH); 126.3 (C-1); 127.4 (2C, 2× CH); 127.9 (CH); 128.5 (2C, 2× CH); 132.2 (C-10); 137.2 (C); 137.8 (C); 156.9 (C-3); 218.5 (C=O). MS *m*/*z* (%) 416 (100, [M+H]^+^).

### 4.2. Cell CUlture and CHemicals

The utilized cell lines (HeLa, MDA-MB-231, MCF-7, and NIH/3T3) were obtained from ECACC (European Collection of Cell Cultures, Salisbury, UK), except for SiHa cells which were obtained from ATCC (American Tissue Culture Collection, Manassas, VA, USA). All cell lines were cultured in Eagle’s Minimum Essential Medium (EMEM) at 37 °C in a humidified atmosphere with 5% carbon dioxide. The medium was supplemented with 10% fetal bovine serum (FBS), 1% non-essential amino acid solution, and 1% penicillin, streptomycin, and amphotericin B mixture. All cell culture mediums and supplements were obtained from Lonza Group Ltd. (Basel, Switzerland). Chemicals for the described in vitro experiments were purchased from Merck Ltd. (Budapest, Hungary) unless stated otherwise.

### 4.3. Determination of Antiproliferative Activity (MTT Assay)

The antiproliferative activity of the presented compounds were evaluated against a panel of human gynecological cancer cell lines. MCF-7 and MDA-MB-231 cell lines were derived from breast cancers, while HeLa and SiHa cell lines originated from cervical cancers of different pathological backgrounds. Non-cancerous human fibroblast cells (NIH/3T3) were used exclusively to assess cancer selectivity of the two azidomethyl compounds.

Cancer cells were seeded onto a 96-well microplate for the proliferation assay at a density of 5000 cells/well. After 24 h of incubation, 200 μL of new medium containing the test compounds at 10 or 30 µM concentrations was added.

Following incubation for 72 h at 37 °C in a humidified atmosphere containing 5% CO_2_, cell viability was assessed by adding 20 μL of 5 mg/mL 3-(4,5-dimethylthiazol-2-yl)-2,5-diphenyltetrazolium bromide (MTT) solution. After 4 h of incubation, the yellow MTT solution was converted to violet crystals by mitochondrial reductases in viable cells. Subsequently, the medium was removed, and the formazan crystals were dissolved in 100 μL of DMSO with shaking at 37 °C for 60 min.

Absorbance of the reduced MTT solution was measured at 545 nm using a microplate reader, with untreated cells serving as the negative control [31]. In the case of active compounds (i.e., >50% cell growth inhibition at 10 μM), the assay was repeated with a series of dilutions, and sigmoidal dose–response curves were fitted to the obtained data. The IC_50_ values, representing the concentration at which cell proliferation was reduced by 50% compared with the untreated control, were calculated using GraphPad Prism 5 (GraphPad Software, San Diego, CA, USA). Each in vitro experiment was conducted on two microplates with a minimum of five parallel wells. Stock solutions of the test substances (10 mM) were prepared in DMSO, with the highest DMSO concentration in the medium not exceeding 0.3%, which did not significantly affect cell proliferation. Cisplatin was used as a reference agent.

### 4.4. Propidium Iodide-Based Cell Cycle Analysis

Cell cycle analysis was conducted to investigate the mechanism of action of azidomethyl compounds in human breast cancer cell lines. Specifically, MDA-MB-231 cells were seeded onto 24-well plates at a density of 80,000 cells per well. The cells were treated with two concentrations of **16AABE** (0.5 or 1 μM) and **16BABE** (2 or 4 μM), respectively, for 24 h.

After treatment, the cells were washed with phosphate-buffered saline (PBS) and harvested using trypsin. The harvested cells were combined with the supernatants and PBS from the washing process. Subsequently, centrifugation at 1700 rpm for 5 min at room temperature was performed, followed by resuspending the cell pellets in a DNA staining solution. The DNA staining solution consisted of 10 μg/mL propidium iodide (PI), 0.1% Triton-X, 10 μg/mL RNase A, and 0.1% sodium citrate dissolved in PBS. The resuspended cells were then incubated in dark at room temperature for 30 min.

At least 20,000 events per sample were analyzed using a FACSCalibur (BD Biosciences, Franklin Lakes, NJ, USA) flow cytometer to assess the DNA content. Data obtained were analyzed using the ModFit LT 3.3.11 software (Verity Software House, Topsham, ME, USA). Untreated cells served as the control, and the hypodiploid (subG1) phase indicated the apoptotic cell population [21].

### 4.5. Tubulin Polymerization Assay

Following the manufacturer’s instructions, a tubulin polymerization assay kit (Cytoskeleton Inc., Denver, CO, USA) was employed to assess cell-independent direct effects of **16AABE** and **16BABE** on tubulin polymerization in vitro. Initially, 10 μL of a 500 μM solution of the desired compound was added to a UV-transparent microplate prewarmed to 37 °C. Positive control samples containing 10 μL of 10 μM paclitaxel, as well as untreated controls with general tubulin buffer (80 mM PIPES pH 6.9, 2 mM MgCl_2_, 0.5 mM EGTA) were also prepared. Next, 100 μL of a 3.0 mg/mL tubulin solution dissolved in polymerization buffer (80 mM PIPES pH 6.9, 2 mM MgCl_2_, 0.5 mM EGTA, 1 mM GTP, 10.2% glycerol) was added to each sample present in separate wells of a 96-well plate. The plate was immediately placed in an ultraviolet spectrophotometer (SPECTROstarNano, BMG Labtech, Ortenberg, Germany) prewarmed to 37 °C. A 60-min kinetic reaction was initiated, during which the absorbance was measured at 340 nm every minute to evaluate the effects of the test compounds. The tubulin polymerization curve was constructed by plotting the optical density against time. Maximum reaction rate (Vmax; Δabsorbance/min) was calculated based on the highest difference in absorbance observed over three consecutive time points on the kinetic curve.

### 4.6. Migration Assay

As previously described, MCF-7 cell suspension was prepared in a supplemented EMEM. The cells were then seeded onto 12-well plates using specialized silicone inserts (Ibidi GmbH, Grafelfing, Germany) at a concentration of 25,000 cells per well. The silicone inserts were gently removed after an overnight incubation, and the cells were washed with PBS. Subsequently, the cells were subjected to a wound healing assay by treating them with low concentrations of the test compounds (1.5 and 3 μM) prepared in EMEM medium with reduced serum content (2% FBS).

Antimigratory effect of the test compounds was assessed by measuring the size of cell-free areas. Images of the cell monolayer were captured at 0, 24, and 48 h using the QCapture Pro 6.0 software. Based on the captured images, the size of cell-free areas was determined using the ImageJ 1.53e software (National Institutes of Health, Bethesda, MD, USA).

### 4.7. Invasion Assay

To assess the impact of our test compounds on the invasion capacity of malignant MDA-MB-231 cells, we employed Boyden chambers equipped with a reconstituted membrane that mimics the basement membrane (BD Biosciences, Bedford, MA, USA). Treated cells were carefully pipetted onto the hydrated membranes in the upper chamber. In the lower chamber, EMEM supplemented with 10% FBS served as a chemoattractant. After a 24 h incubation period, the supernatants were removed, and non-invading cells on the upper side of the membrane were gently wiped using a cotton swab. The membrane was then rinsed twice with PBS and fixed with ice-cold 96% ethanol. Subsequently, invading cells were stained with 1% crystal violet dye solution for 30 min in the dark at room temperature. Multiple images (at least three per insert) were captured using a Nikon Eclipse TS100 microscope (Nikon Instruments Europe, Amstelveen, The Netherlands). Finally, invading cells were quantified and compared with untreated control samples.

### 4.8. Determination of Estrogenic Activity

T47D human breast adenocarcinoma cells expressing endogenous estrogen receptor (ERα), modified with an estrogen-responsive luciferase (Luc) reporter gene (T47D-KBluc, obtained from ATCC, Manassas, VA, USA) were used to assess the estrogenic activity of **16AABE** and **16BABE** [32]. Cells were maintained in phenol red-free MEM with 2 mM L-glutamine, 1 g/L glucose, 10% FBS and penicillin–streptomycin antibiotics. Before testing the compounds’ effect, cells were maintained in the medium described above, supplemented with 10% charcoal dextran-treated FBS for at least six days. Cells were seeded onto a 96-well white flat bottom plate (Greiner Bio-One, Mosonmagyaróvár, Hungary) at a density of 50,000 per well in 200 µL of the medium above, and were allowed to attach for 72 h. Then the indicated concentrations of the test compounds or the reference agent 17β-estradiol were added (less than 0.1% DMSO in the final concentration). Plates were incubated at 37 °C in a humidified 5% CO_2_ incubator before measuring luciferase activity. After 24 h of incubation, the dosing media was removed entirely, and 30 μL of One-Glo firefly luciferase reagent (Promega, Madison, WI, USA) per well was added to the plate, followed by incubation for 3 min at room temperature according to the manufacturer’s protocol, and then the luminescence signal was quantified (FLUOstar Optima, BMG Labtech, Ortenberg, Germany).

### 4.9. Statistical Analysis

Statistical data analysis was conducted using the GraphPad Prism 5 software (GraphPad, San Diego, CA, USA). One-way analysis of variance (ANOVA) was employed, followed by the Dunnett post-test, to assess the significance of the observed differences. Data are expressed as mean values ± standard error of the mean (SEM).

## 5. Conclusions

In conclusion, our findings provide compelling experimental evidence to support the relevance of 16-azidomethyl-estrone analogs as potential drug candidates with anticancer properties. The observed tumor-selective antiproliferative and antimetastatic effects, combined with their ability to induce cell cycle disturbances and exhibit tumor selectivity, highlight the promising prospects of these compounds as innovative anticancer agents. Furthermore, their potent antimigratory and anti-invasive properties are exerted below their growth-inhibitory concentrations. The tested compounds substantially increased the polymerization of tubulin, which may be the basis of their actions. Since **16AABE** and **16BABE** possess estrogenic activity, their further development seems rational for treating hormone-independent malignancies.

## Figures and Tables

**Table 1:** Tumor selectivity indices of **16AABE** and **16BABE** expressed as the ratio of IC_50_ values obtained against cancer cells and fibroblasts.

Cancer Cell Line	$\frac{{IC}_{50}\;of\;Cancer\;Cell\;Line\;(\mu M)}{{IC}_{50}\;of\;NIH/3T3\;(\mu M)}$	
	16AABE	16BABE
HeLa	0.369	0.243
SiHa	0.302	0.203
MCF-7	0.230	0.166
MDA-MB-231	0.347	0.429

## Data Availability

Data are available upon request.

## Supplementary Materials

The supporting information can be downloaded at: https://www.mdpi.com/article/10.3390/ijms241813749/s1.
