# Supplementary material for: Antiproliferative and Antimetastatic Properties of 16-Azidomethyl Substituted 3-O-Benzyl Estrone Analogs
Source: Int J Mol Sci. 2023 Sep 6;24(18):13749. doi: 10.3390/ijms241813749 (PMC10531082; doi:10.3390/ijms241813749)

Antiproliferative and Antimetastatic Properties of 16-Azidomethyl Substituted 3-*O*-Benzyl Estrone Analogs

Seyyed Ashkan Senobar Tahaei, Ágnes Kulmány, Renáta Minorics, Anita Kiss, Zoltán Szabó, Péter Germán, Gábor J. Szebeni, Nikolett Gémes, Erzsébet Mernyák and István Zupkó

Supplementary Materials

Table S1. Antiproliferative properties of the investigated molecules.

Comp.	Conc. (μM)	Growth inhibition (%) ± SEM and calculated IC_{50} values (μM) [95% Confidence Interval (μM)]				
		**HeLa**	**SiHa**	**MDA-MB-231**	**MCF-7**	**NIH/3T3**
**1**	10	33.97 ± 0.33	<20*	34.07 ± 0.27	38.82 ± 0.81	n.d.
	30	93.10 ± 0.21	89.93 ± 0.20	92.92 ± 0.09	92.64 ± 0.22	
**2**	10	29.15 ± 1.06	20	45.06 ± 1.69	20.22 ± 2.19	n.d.
	30	55.54 ± 2.06	43.16 ± 2.58	64.22 ± 1.39	51.86 ± 1.85	
**3**	10	24.96 ± 2.26	<20	33.50 ± 2.92	27.33 ± 1.28	n.d.
	30	93.35 ± 0.32	86.46 ± 0.36	89.05 ± 0.90	92.55 ± 0.18	
**4**	10	26.63 ± 1.78	<20	25.14 ± 2.83	<20	n.d.
	30	52.89 ± 0.49	31.91 ± 0.69	57.32 ± 0.98	52.42 ± 2.50	
**16AABE**	10	93.22 ± 0.92	90.10 ± 0.66	93.53 ± 0.17	91.73 ± 0.71	<20
	30	93.96 ± 0.23	91.48 ± 0.55	95.11 ± 0.31	93.85 ± 0.22	98.29 ± 0.41
	IC_{50}	5.01 [4.59-5.46]	4.10 [3.26-5.06]	4.72 [3.47-6.43]	3.13 [2.19-4.42]	13.59 [10.54-16.83]
**16BABE**	10	93.40 ± 0.13	90.73 ± 0.53	93.65 ± 0.24	92.94 ± 0.37	<20
	30	94.33 ± 0.32	91.08 ± 0.55	95.03 ± 0.23	93.79 ± 0.40	94.79 ± 1.63
	IC_{50}	4.60 [4.19-5.02]	3.85 [3.48-4.26]	8.13 [6.07-10.85]	3.15 [2.60-3.85]	18.93 [16.13-22.22]
**cisplatin#**	10	42.61 ± 2.33	86.84 ± 0.50	20.84 ± 0.81	53.03 ± 2.29	91.80 ± 0.39
	30	99.93 ± 0.26	90.18 ± 1.78	74.47 ± 1.20	86.90 ± 1.24	93.68 ± 0.20
	IC_{50}	12.43	7.84	19.13	5.78	2.70

*: The inhibition value is less than 20% and not given numerically.

#: Values from reference [17].

Figure S1. Representative concentration-response curves of test substances 16AABE and 16BABE.

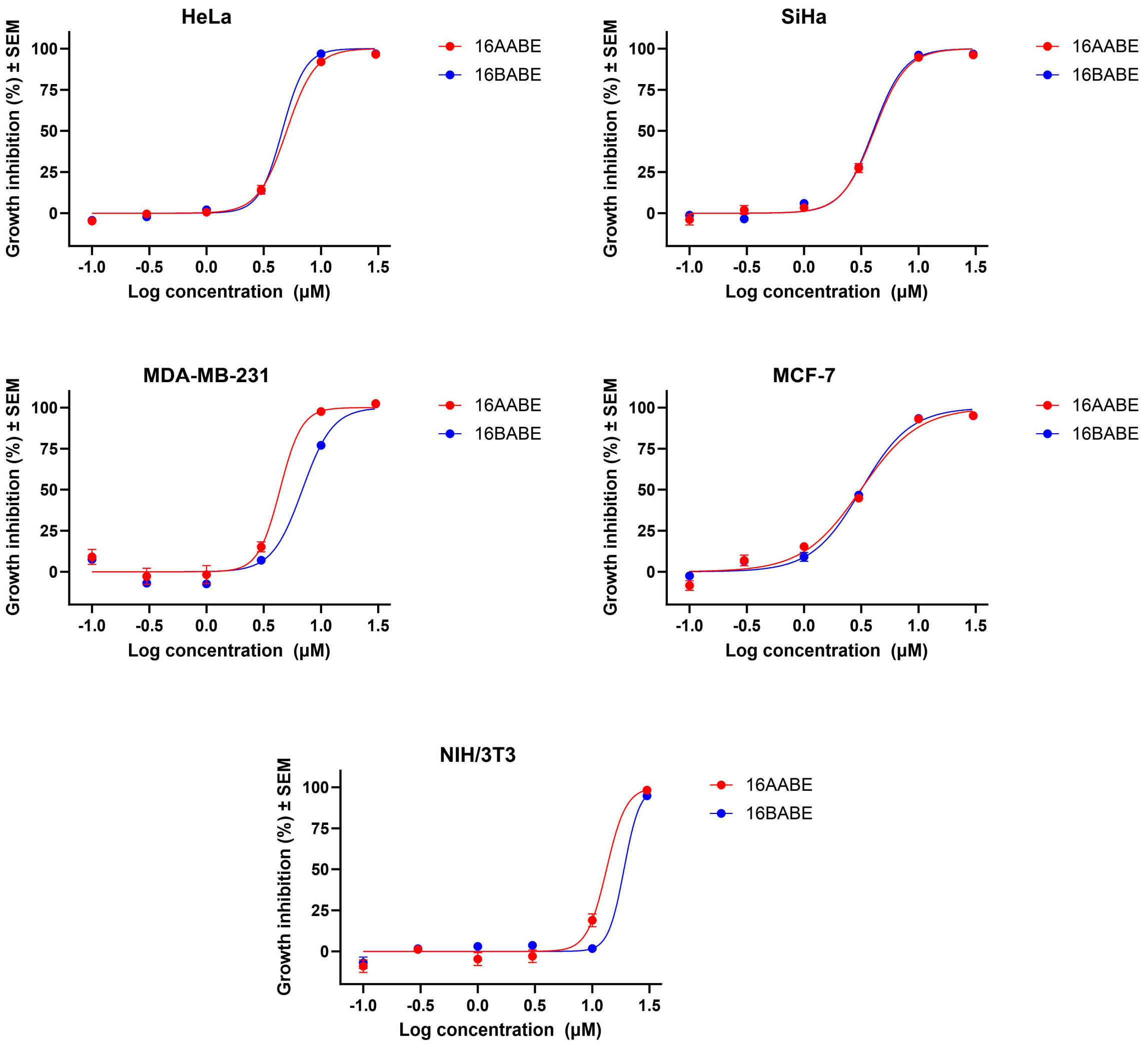

Figure S2. ^{1}H and ^{13}C NMR spectra of 16BABE.

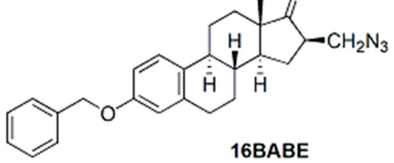

16BABE

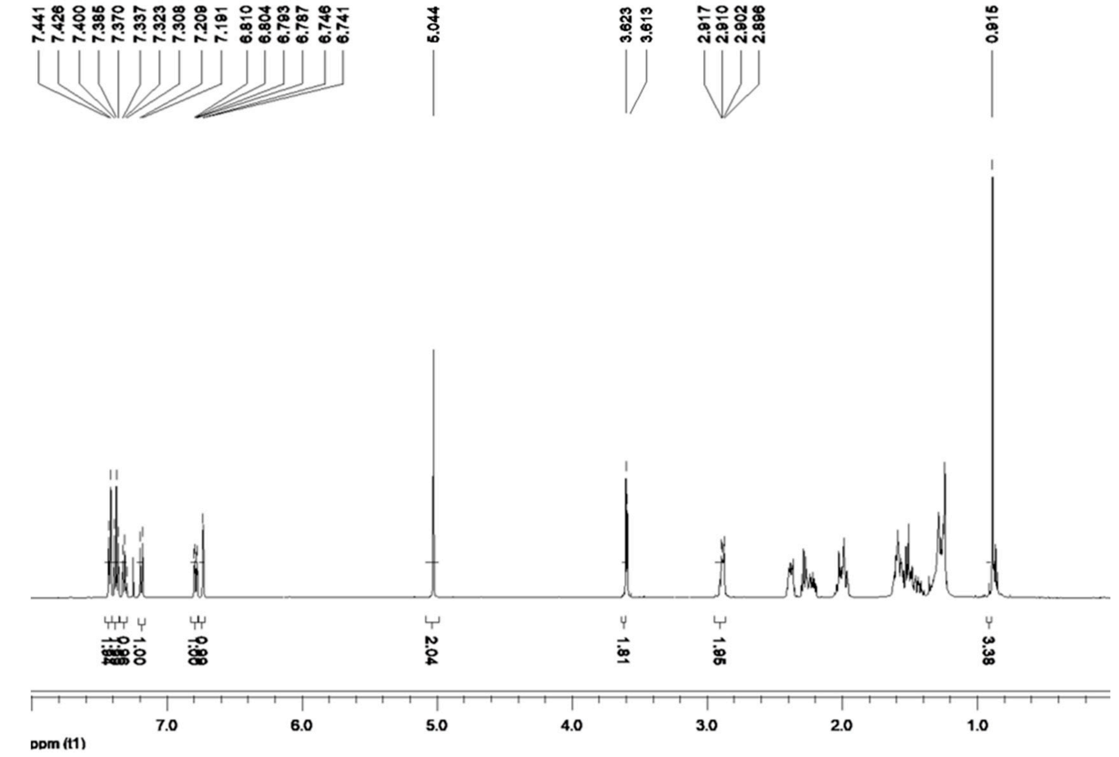

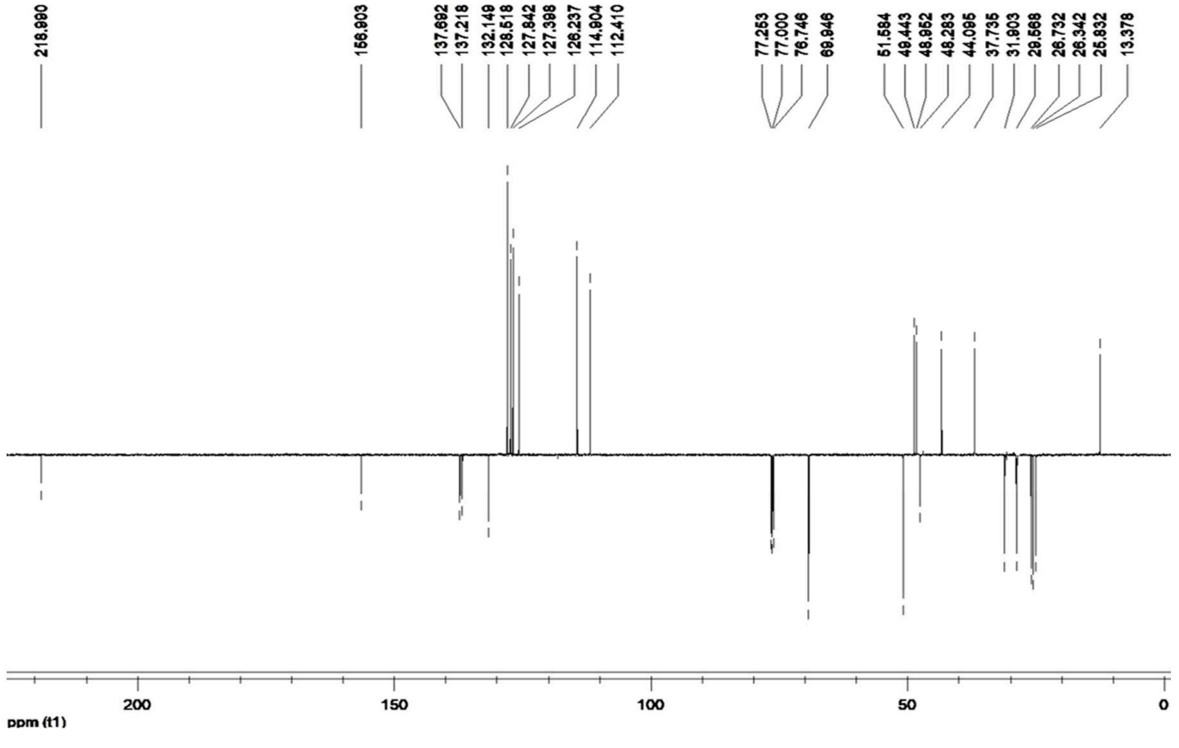

Figure S3. ^{1}H and ^{13}C NMR spectra of 16AABE.

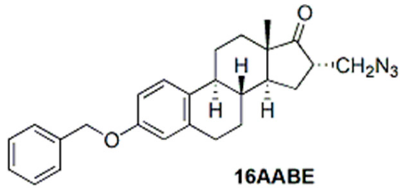

16AABE

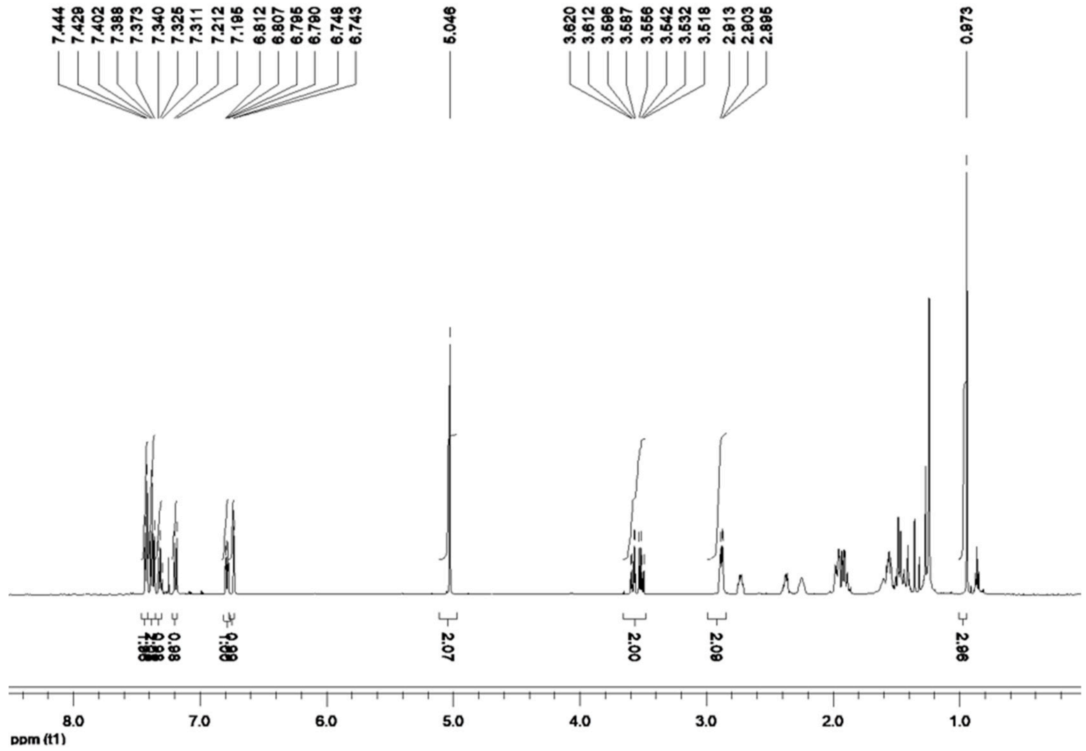

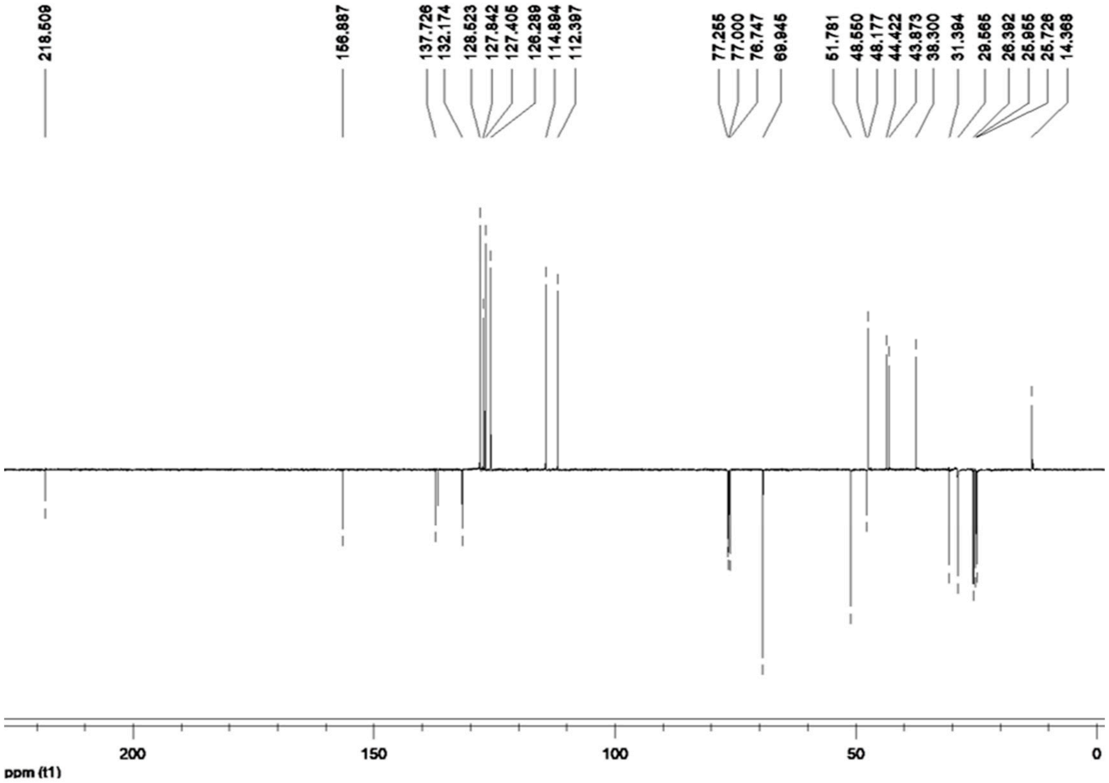

Supplement: Supplementary file 1 [file ijms-24-13749-s001.zip › ijms-2543473-supplementary.pdf]
